# Pediatric pulmonary infection caused by oral obligate anaerobes: Case Series

**DOI:** 10.3389/fped.2023.1226706

**Published:** 2023-09-01

**Authors:** Lai Zhijun, Yang Wenhai, Zeng Peibin, Luo Qingming

**Affiliations:** Department of Pediatric Intensive Care Unit, Dongguan Children's Hospital Affiliated to Guangdong Medical University, Dongguan, China

**Keywords:** obligate anaerobes, lung infection, children, next-generation sequencing, oral hygiene

## Abstract

**Background:**

Pneumonia is quite common in people with chronic bedridden, severe malnutrition and underlying diseases of cerebral palsy. Although poor oral hygiene and inadequate airway protection are risk factors, case reports of childhood pneumonia caused by oral obligate anaerobes are rare.

**Introduction:**

We reported 4 cases of oral anaerobic pneumonia and empyema diagnosed by the pediatric intensive care unit (PICU) of our hospital.

**Discussion:**

No bacteria were detected in sputum bacterial culture, pleural water bacterial culture and blood culture of the four children. Considering that multiple sputum cultures were negative, the pleural effusion and bronchoalveolar lavage fluid were subjected to next-generation sequencing (NGS) to identify the pathogen causing pneumonia. The results found oral obligate anaerobes represented by *Parvimonas micra* and *Porphyromonas gingivalis*. After identifying the pathogenic bacteria, we changed to piperacillin tazobactam combined with metronidazole for anti-infection treatment, and the pneumonia in the above patients was improved. In addition, all four patients had different basic medical histories, and long-term bed rest, severe malnutrition, poor oral hygiene and insufficient airway protection were all high risk factors for oral anaerobic pneumonia in these children.

**Conclusion:**

Oral obligate anaerobes are one of the pathogens to consider for pneumonia in the elderly, but they may be easily overlooked in pediatric groups. Therefore, when receiving children with high-risk factors, we should be alert to the possibility of oral obligate anaerobic bacteria infection. Educating family members to pay attention to children's oral hygiene plays an important role in preventing oral obligatory anaerobic bacteria pneumonia. NGS can be used as a rapid diagnostic method when sputum culture cannot distinguish between pathogens.

## 1. Background

Oral obligate anaerobes are important pathogens of pneumonia in the elderly, but case reports of obligate anaerobes-associated pneumonia in children are rare. Oral obligate anaerobes include *Parvimonas micra* and *Porphyromonas gingivalis*, which can be detected in oral dental plaque (1, 2). There have been case reports of *Parvimonas micra* or *Porphyromonas gingivalis* causing (3), phlebitis (4), abdominal abscess (5) and intracranial abscess (6). As one of the common pathogenic bacteria of aspiration pneumonia in the elderly population (7, 8), anaerobic lung infection is associated with oral secretion aspiration, poor oral hygiene conditions and inadequate airway protection (9).

Anaerobic pneumonia is relatively rare in normal children, but some special children have high risk factors for anaerobic pneumonia. Clinical antimicrobial treatment of community-acquired pneumonia in children caused by bacteria does not cover anaerobic bacteria, so uncommon pathogens often need to be considered in the case of poor conventional anti-infective treatment. Because oral obligate anaerobes are difficult to define by bacterial culture, early adoption of NGS can facilitate rapid diagnosis to initiate appropriate anti-infective treatment.

## 2. Methods of NGS

### 2.1. Sample processing and DNA extraction

1.5–3 ml BALF sample from patient was collected according to standard procedures. 1.5 ml microcentrifuge tube with 0.6 ml sample and 250 µl 0.5 mm glass bead were attached to a horizontal platform on a vortex mixer and agitated vigorously at 2800–3200 rpm for 30 min. Then 7.2 µl lysozyme was added for wall-breaking reaction. 0.3 ml sample was separated into a new 1.5 ml microcentrifuge tube and DNA was extracted using the TIANamp Micro DNA Kit (DP316, TIANGEN BIOTECH) according to the manufacturer's recommendation. The extracted DNA specimens were used for the construction of DNA libraries (10).

### 2.2. RNA library construction and sequencing

Microbial RNA was extracted from BALF samples using a QIAamp Viral RNA Mini Kit (Qiagen, 250, Germany) according to the manufacturer's recommendations. RNA was fragmented, reverse-transcribed, end-repaired, ligated to adapters, and amplified by PCR.

### 2.3. Construction of DNA libraries and sequencing

Then, DNA libraries were constructed through DNA-fragmentation, end-repair, adapter-ligation and PCR amplification. Agilent 2,100 was used for quality control of the DNA libraries. Quality qualified libraries were pooled, DNA Nanoball (DNB) was made and sequenced by BGISEQ-50 /MGISEQ-2000 platform (11).

### 2.4. Bioinformatic analysis

High-quality sequencing data were generated by removing low-quality reads, followed by computational substraction of human host sequences mapped to the human reference genome (hg19) using Burrows–Wheeler Alignment (12). The remaining data by removal of low-complexity reads were classified by simultaneously aligning to Pathogens metagenomics Database (PMDB), consisting of bacteria, fungi, viruses and parasites. The classification reference databases were downloaded from NCBI (ftp://ftp.ncbi.nlm.nih.gov/genomes/). RefSeq contains 4,945 whole genome sequence of viral taxa, 6,350 bacteral genomes or scaffolds, 1,064 fungi related to human infection, and 234 parasites associated with human diseases.

## 3. Case introduction

From 2020 to 2022, four children with pneumonia diagnosed as oral obligate anaerobic bacteria were admitted to the PICU of our hospital.

Three of the four children were male and 1 female. Their mean age was 10.3 years (age range from 8 to 13 years). All patients had different underlying diseases (Down syndrome (*n* = 1), cerebral palsy (*n* = 2), hypophrenia (*n* = 1), severe malnutrition (*n* = 4), nasal tube feeding (*n* = 3)) (Table 1). All four children had poor oral hygiene, more tartar, and different degrees of dental caries.

**Table 1:** Age, gender, underlying disease, nutritional status, feeding practices, and prognosis.

Case	Age	Gender	Oral hygiene	Underlying disease	Nutriture	Feeding patterns	Prognosis
1	8	M	Poor	Down's syndrome	Severe malnutrition	Eat through the mouth	Recurred and discharge
2	13	M	Poor	Cerebral palsy	Severe malnutrition	Nasal feeding tube feeding	Recurred and discharge
3	11	M	Poor	Cerebral palsy	Severe malnutrition	Nasal feeding tube feeding	Recurred and discharge
4	9	F	Poor	Hypophrenia	Severe malnutrition	Eat through the mouth	Recurred and discharge

**Figure 1:**
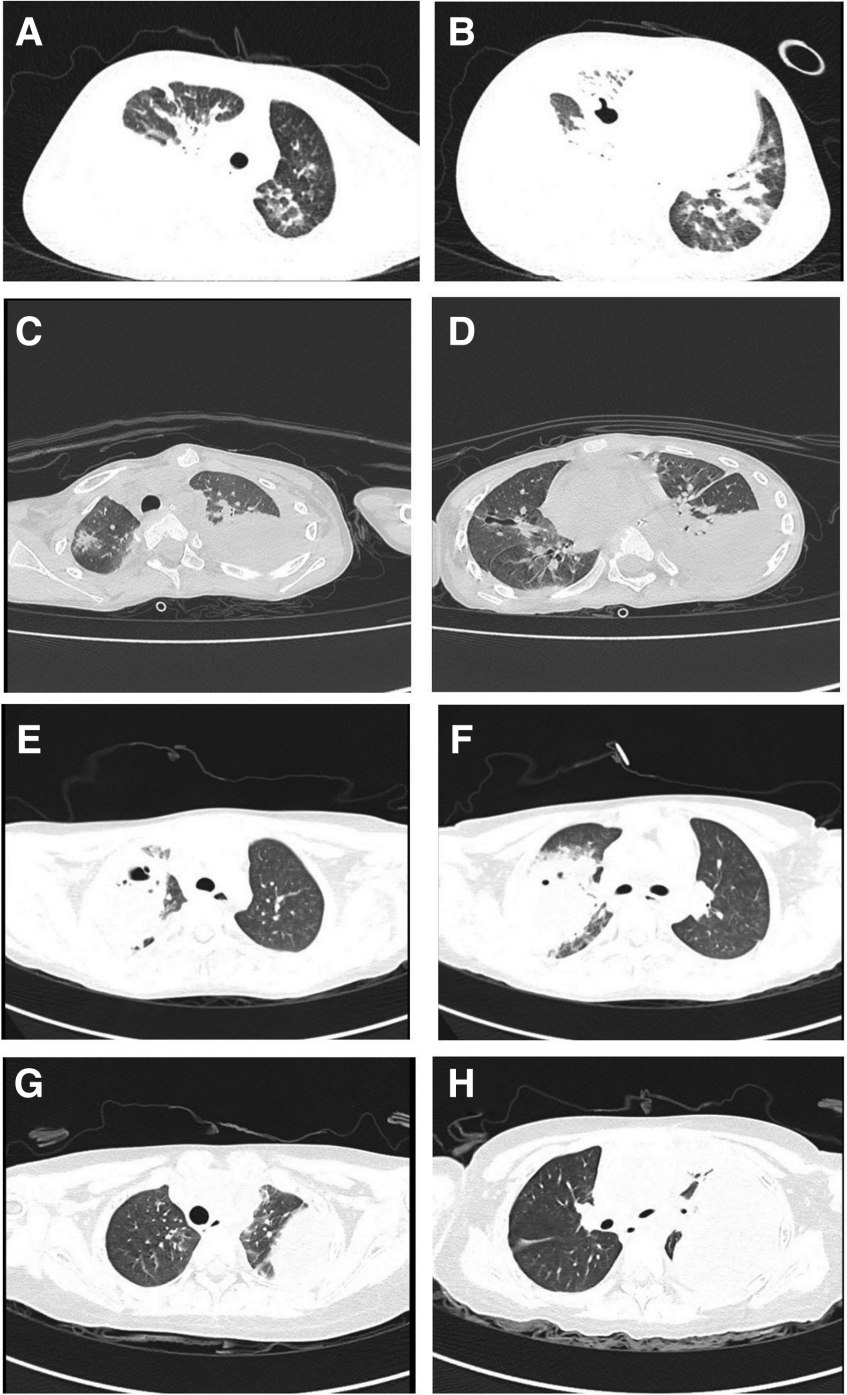
Pulmonary CT findings: (**A,B**) are the CT of case 1. (**C,D**) are the CT of case 2. (**E,F**) are the CT of case 3. (**G,H**) are CT of case 4.

**Figure 2:**
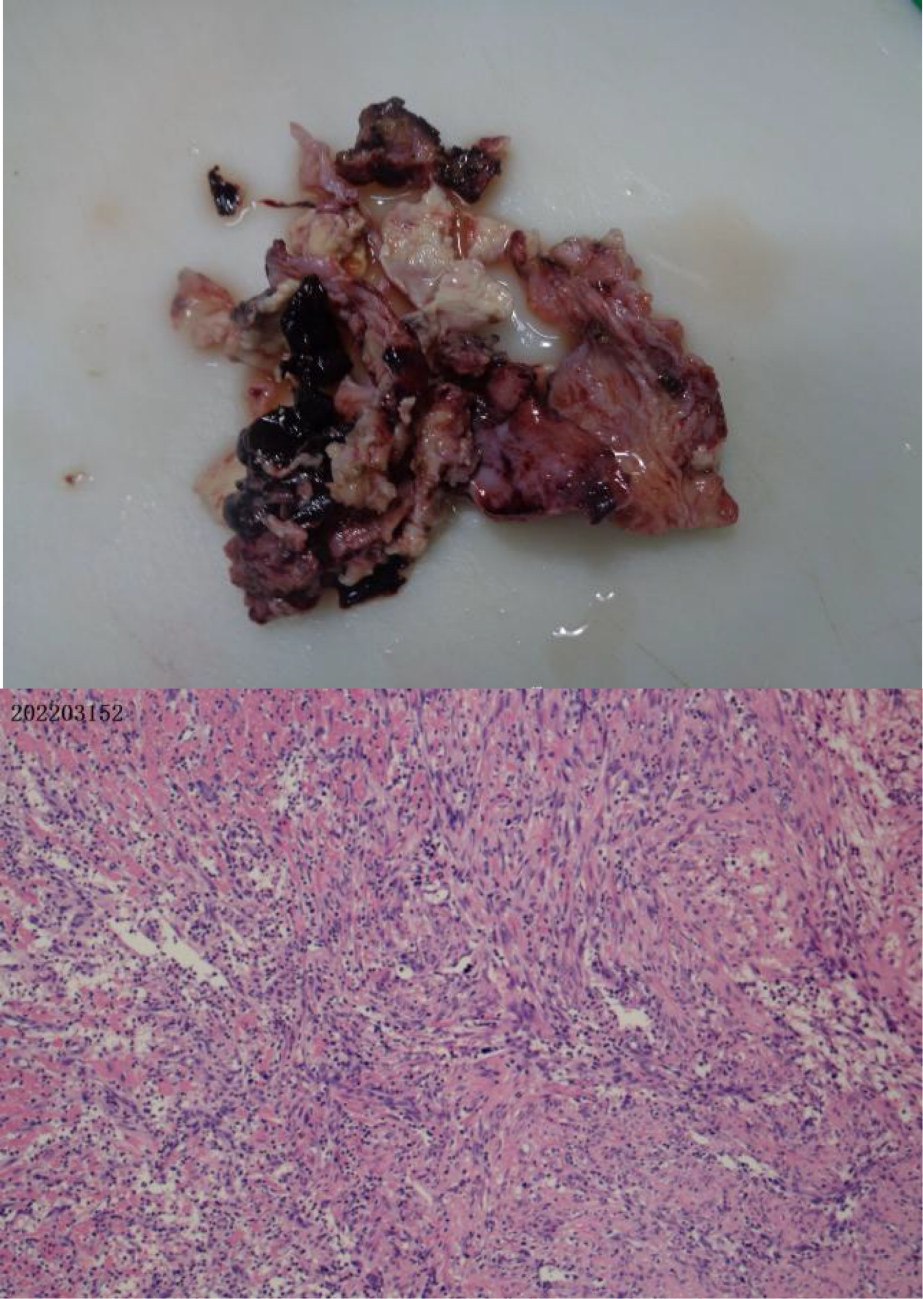
Plot of the lung damaged tissue and pathological sections in case 4. Extensive leukocyte infiltration was seen in the pathological sections.

Clinical symptoms included fever (*n* = 4), cough (*n* = 4), shortness of breath (*n* = 2), haemoptysis (*n* = 1), and respiratory failure (*n* = 1). Only one child required mechanical ventilation during hospitalization, and three others required only low-flow oxygen support. Finally, the four children were improved and discharged (Table 2). All patients had computed tomography scans showing pleural effusion (*n* = 4) and lung abscess (*n* = 4) (Figure 1). One of the children underwent lung necrosis tissue resection, and massive leukocyte infiltration was seen in the removed lung histopathological sections (Figure 2).

**Table 2:** Clinical symptoms and respiratory support status.

Case	Fever	Cough	Anhelation	Hemoptysis	Respiratory failure	Pleural effussion	Pulmonary abscess	Mechanical ventilation
1	+	+	+	+	+	A large amount	+	+
2	+	+	−	−	−	A small amount	+	−
3	+	+	−	−	−	A large amount	+	−
4	+	+	+	−	−	A large amount	+	−

In terms of etiology detection, all the children with sputum bacterial culture and blood culture did not detect bacteria. Three children underwent thoracentesis during hospitalization and sent pleural effusion samples for NGS to identify the pathogen. Another child underwent fiberoptic bronchoscopic alveolar lavage and sent this child's alveolar lavage fluid for NGS. NGS tests in 4 children showed that *Porphyromonas* (*n* = 4), *Parvimonas micra* (*n* = 3), *Fusobacterium* (*n* = 2), *Streptococcus* (*n* = 2), *Pyramidobacter piscolens* (*n* = 1), *Peptostreptococcus stomatis* (*n* = 1), *Filifactor alocis* (*n* = 1), *Prevotella oris* (*n* = 1), *Tannerella forsythia* (*n* = 1), *Bacteroides heparinolyticus* (*n* = 1), *Solobacterium moorei* (*n = *1), *Dialister pneumosintes* (*n* = 1), and *Catonella morbi* (*n* = 1) (Table 3).

**Table 3:** The reads of pathological detection in NGS report.

Case	1	2	3	4
Sample type	Pleural effussion	Pleural effussion	The alveolar lavage fluid	Pleural effussion
*Porphyromonas gingivalis*	1,868	25	17,879	85
*Parvimonas micra*	2,031	−	37,964	138
*Catonella morbi*	1,139	−	−	−
*Fusobacterium nucleatum*	1,100	4	−	−
*Streptococcus constellatus*	440	−	24	−
*Pyramidobacter piscolens*	−	−	40,842	−
*Peptostreptococcus stomatis*	−	−	10,996	−
*Filifactor alocis*	−	−	−	98
*Prevotella oris*	−	−	4,098	−
*Tannerella forsythia*	−	−	−	60
*Bacteroides heparinolyticus*	−	−	15,298	−
*Solobacterium moorei*	−	−	15,781	−
*Dialister pneumosintes*	−	−	9,970	−

## 4. Discussion

Poor oral hygiene has been shown to be associated with obligate anaerobic bacteria in the lower respiratory tract by Ryosuke Hata et al. (9). The plaque biofilm on the tooth surface is a large reservoir of bacteria and in specific circumstances these bacteria may cause lower respiratory tract infection (13). These oral colonizing bacteria are also important pathogens causing aspiration pneumonia (14). Although Ryosuke Hata et al. targeted the elderly group and did not conduct a studies of children, our reported cases were similar to the elderly group in the above study. They also lack the ability to maintain good oral hygiene independently, and they also have airway protection or swallowing disorders. All of the above reasons are the high risk factors of oral obligatory anaerobic bacteria pneumonia in this group.

**Figure 3:**
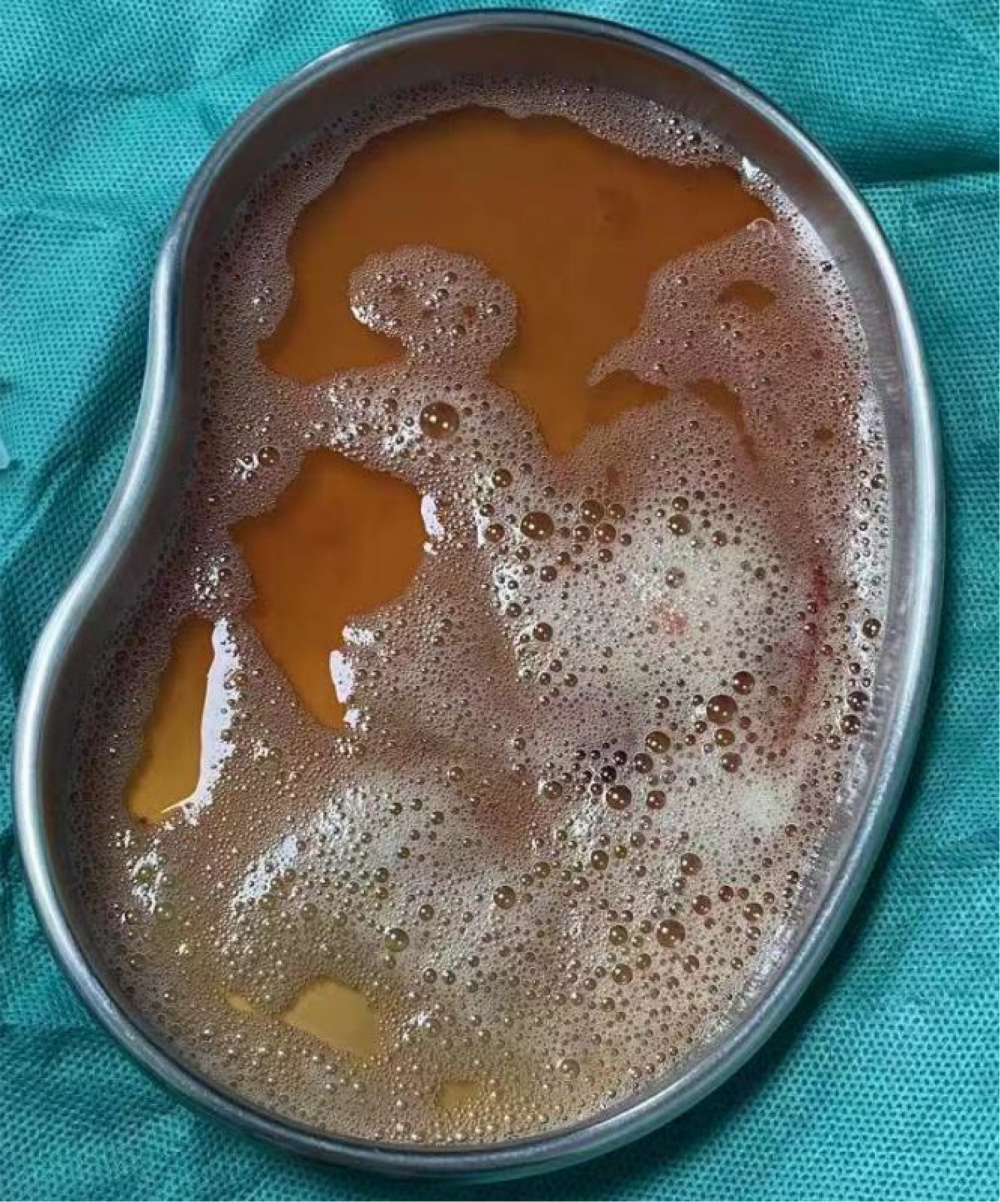
The pleural effussion of case 4. The patient's pleural effusion was brownish.

In our reported cases, the pathogens with the highest percentage of detection were *Porphyromonas gingivalis* as well as *Parvimonas micra*. Both are obligate anaerobes with oral colonization, and they both need to reproduce in a strictly sterile environment. In general, obligate anaerobic bacteria can hardly survive in an oxygenated environment in the lungs. But because the host has long-term bed or the cough reflex drops in the lungs, it may create a local hypoxia environment, creating conditions for the growth of obligate anaerobic bacteria to thrive. Before all patients underwent CT, we communicated with the parents about the condition, and none of the parent did consent for chest enhanced CT, and therefore the results of chest enhanced CT were lacking. The CT examinations of all four children were suggestive of varying degrees of pleural effusion. We all know that the pleural cavity is originally a confined space, which is conducive to the growth of obligate anaerobic bacteria. We kept samples of diseased lung tissue, but limited by the high cost of NGS examination, we failed to conduct NGS examination of diseased tissue (Figure 2). Pleural infection caused by an anaerobe is the cause of a large pleural effusion. The pleural effusion extracted in case 4 in our report is brownish (Figure 3).

In our report, there were two cases each of left and right pleural effusion. Although it is generally believed that aspiration pneumonia is more likely in the right lung, not all cases developed infection in the right lung. We considered that this may be associated with maintaining different positions in different patients. A prolonged left lateral position may be more likely to cause aspiration pneumonia in the left lung.

All sputum and blood cultures during hospitalization of the children in this case report were negative. Bacterial cultures of pleural effusion were performed in three children, but these bacterial cultures were negative. This is related to the specific growth conditions of obligate anaerobes, so it is often difficult to diagnose anaerobic infections clinically by bacterial culture. We found that NGS has been applied in the diagnosis of encephalitis (15) and infectious pancreatitis (16). Therefore, when the initial stage of anti-infection treatment effect is not satisfactory, We performed NGS examinations to clarify the etiology. When we have obtained the NGS report, we thought there were 2 main reasons support our consideration of anaerobe-associated pneumonia: ① These patients were treated with piperacillin sulbactam/piperacillin tazobactam, but the effect of treatment was unsatisfy. This suggests that the infectious source is not within the coverage of this antibiotic. ② In the NGS report, the relevant pathogen was clearly detected. As we knew, piperacillin sulbactam/piperacillin tazobactam is unsatisfy in treating infections caused by these pathogens.

We immediately switched to metronidazole (7.5 mg/kg q8h) combined with piperacillin tazobactam (4:1,50 mg/kg q8h) after confirming the NGS results. After adjusting the anti-infection treatment regimen, all the children improved and were eventually discharged. NGS as a novel detection method helps detecting special pathogens. Despite the high price of NGS, it is still a clinically important test.

## 5. Conclusion

The elderly and the immunocompromised group were previously considered to be at high risk for anaerobic pneumonia (17, 18), but some special child groups may be easily overlooked (19). Such children often have high-risk factors such as poor oral hygiene, inadequate airway protection, swallowing dysfunction, long-term bed and malnutrition. In treating such children, we need to consider special etiological infections possible if conventional anti-infective treatment is ineffective. NGS may be an effective means for early and rapid diagnosis. After identifying the cause, the targeted anti-infection regimen is critical to the prognosis of the child.

For families with such special children, health care workers need adequate health education to develop good awareness of the disease. Grasping the ability of effective oral care and cleaning has an irreplaceable role in reducing the occurrence of anaerobic bacteria pneumonia.

## Data Availability

The original contributions presented in the study are included in the article/supplementary material, further inquiries can be directed to the corresponding author/s.
